# Outcomes after elective inguinal hernia repair with mesh performed by associate clinicians *versus* medical doctors in Sierra Leone: 5-year follow-up of a randomized clinical trial

**DOI:** 10.1093/bjs/znaf221

**Published:** 2025-12-10

**Authors:** Thomas Ashley, Hannah F Ashley, Andreas Wladis, Pär Nordin, Michael Ohene-Yeboah, Isaac O Smalle, Jessica H Beard, Jenny Löfgren, Håkon A Bolkan, Alex J van Duinen

**Affiliations:** Department of Surgery, University of Sierra Leone Teaching Hospital Complex (USLTHC), Connaught Hospital, Freetown, Sierra Leone; CapaCare, Masanga, Sierra Leone; CapaCare, Masanga, Sierra Leone; The Lakes Medical Practice, Penrith, UK; Department of Biomedical and Clinical Sciences, Linköping University, Linköping, Sweden; Department of Surgical and Perioperative Sciences, Umeå University, Umeå, Sweden; Department of Surgery, University of Ghana Medical School, Korle Bu, Accra, Ghana; Department of Surgery, University of Sierra Leone Teaching Hospital Complex (USLTHC), Connaught Hospital, Freetown, Sierra Leone; Department of Surgery, College of Medicine and Allied Health Sciences, University of Sierra Leone, Freetown, Sierra Leone; Department of Surgery, Lewis Katz School of Medicine at Temple University, Philadelphia, Pennsylvania, USA; Department of Molecular Medicine and Surgery, Karolinska Institutet, Stockholm, Sweden; CapaCare, Masanga, Sierra Leone; Department of Surgery, St Olav’s Hospital, Trondheim University Hospital, Trondheim, Norway; Institute of Nursing and Public Health, Norwegian University of Science and Technology (NTNU), Trondheim, Norway; CapaCare, Masanga, Sierra Leone; Department of Surgery, St Olav’s Hospital, Trondheim University Hospital, Trondheim, Norway; Institute of Nursing and Public Health, Norwegian University of Science and Technology (NTNU), Trondheim, Norway

## Abstract

**Background:**

Inguinal hernia repair is one of the most performed surgical procedures, but, nevertheless, there is a high unmet need, with over 200 million people worldwide living with an inguinal hernia. The aims of this study were to evaluate 5-year outcomes after anterior mesh inguinal hernia repair, to assess the safety of a training intervention, and to compare the outcomes of patients operated on by a medical doctor (MD) *versus* an associate clinician (AC).

**Methods:**

Adult men with a primary inguinal hernia were included either as training patients or in the randomized trial, with surgical treatment performed by an MD or an AC. Patients were followed up mostly at hospital or at home; questionnaire information was collected and physical examinations were performed. Outcomes of training and trial patients were compared and outcomes of patients who underwent surgeries performed by MDs or ACs during the trial were compared.

**Results:**

In total, 129 patients were included in the training group and 229 patients were included in the randomized trial group. At 5-year follow-up, 288 patients (80.4%) were alive, 40 patients (11.2%) had died, and 30 patients (8.4%) were lost to follow-up. The overall recurrence rate was 5.0% and the all-cause mortality rate was 11.2%. Mortality and recurrence were not significantly different between the training and trial patients or between the patients who underwent surgeries performed by MDs or ACs during the trial.

**Conclusion:**

Long-term outcomes after primary elective inguinal mesh hernia repair indicate that hands-on short-course training can be implemented effectively and that task sharing is safe and effective.

## Teaser

This article presents the 5-year results of an RCT comparing 229 inguinal mesh hernia repairs performed by medical doctors and associate clinicians, as well as for a group of 129 patients who participated in short-course competency-based training in preparation for the trial. Mortality and recurrence were not significantly different between the training and trial patients or between the patients who underwent surgeries performed by MDs or ACs during the trial, demonstrating that hands-on short-course training can be implemented effectively and that task sharing is safe and effective.

## Introduction

An inguinal hernia is a common condition, with an overall prevalence of 7.7%, affecting men more frequently than women (9.6% *versus* 1.3% respectively)^1^. Consequently, inguinal hernia repair is one of the most commonly performed general surgeries worldwide, with 20 million procedures carried out annually^2^. The estimated number of people living with the condition is >200 million and thus the unmet need for hernia surgery is vast^3^. Populations in Africa are particularly underserved^4,5^. Lack of access to inguinal hernia surgery in low-income settings leads to preventable morbidity, mortality, and economic loss^6^.

In sub-Saharan Africa, inguinal hernias are usually repaired using sutured tissue techniques due to financial constraints with regard to mesh and limited training in mesh repair techniques for those who perform surgery^7^. Using mesh to achieve a tension-free inguinal hernia repair significantly reduces the risk of hernia recurrence and has been the preferred method in high-income countries for decades^8,9^. Several studies conducted by the authors, following outcomes until 1 year, have demonstrated that mesh repair is both safe and effective in different locations in sub-Saharan Africa and comparable to that in high-income countries^10–12^. A meta-analysis of adult groin hernia surgery in sub-Saharan Africa reported a pooled mortality rate of 0.6% and a recurrence rate of 1.4%^13^.

In Sierra Leone, inguinal hernia repairs are mainly performed by three cadres of health professionals: specialist surgeons; medical doctors (MDs) with no formal surgical training; and associate clinicians (ACs) with 3 years of formal surgical training^14^. Due to a shortage of MDs, especially in rural areas, a task-sharing programme was introduced that trained Community Health Officers in basic-life saving surgery, including hernia surgeries; this cadre is also referred to as ACs^15^. A single-blind, non-inferiority RCT comparing MDs and ACs performing primary elective inguinal hernia repair on healthy adult men demonstrated that surgical task sharing of elective groin hernia repair with ACs was safe and effective^11^. In addition, standardized short-course competency-based training in mesh inguinal hernia repair ahead of the RCT was shown to be feasible and safe^16^. As most recurrences occur >1 year after surgery, long-term follow-up of these patients is crucial^17^.

The aims of this study were to evaluate 5-year outcomes after anterior mesh inguinal hernia repair, to assess the safety of a training intervention, and to compare the outcomes of patients operated on by an MD *versus* an AC.

## Methods

### Study design

This was a 5-year prospective cohort study that included patients from both the training and the RCT who had undergone mesh repair for an inguinal hernia. Patients were included in the study during three surgical camps organized between October 2017 and February 2018 at Kamakwie Wesleyan Hospital, a first-level mission hospital, in the north-west of Sierra Leone.

### Surgical training programme and materials

Trainees were non-specialist MDs and surgically trained ACs. MDs who participated in the study had completed medical school followed by a 1-year internship, including ≥6 months of experience in general surgery, but had no formal training in surgery. The ACs were Community Health Officers who had completed 3 years of formal surgical training, including sutured groin hernia repairs^15^.

The Ghana Hernia Society previously developed an intensive training course in anterior mesh inguinal hernia repair under local anaesthesia for MDs and surgeons already proficient in suture techniques for inguinal hernia repair. An adapted version of this course was provided for MDs and ACs, which included theory on hernia epidemiology, the Lichtenstein technique, and the administration of local anaesthesia. The theory was followed by hands-on practical training and supervised surgeries. The trainers evaluated each trainee’s competence using the American College of Surgeon’s Groin Hernia Operative Performance Rating System^18,19^. Overall scores of four or five for at least two consecutive operations observed by two consultant surgeons separately were required to pass the course and participate in the RCT.

Of the 13 trainees, 11 reached proficiency. All 129 patients who were operated on during this training course were included in this long-term follow-up. After passing the examination, the trainees continued to perform mesh inguinal hernia repairs in an RCT, where, in total, 230 patients were randomly allocated to an operation performed by an MD or an AC. Clinical outcomes after 1 year have been published previously^11,16^ and the present study describes outcomes 5 years after mesh repair for an inguinal hernia.

### Study participants

Recruitment of study participants was achieved through radio announcements, through communication with community chiefs and leaders, and through communication with staff at nearby peripheral health units. Men aged ≥18 years with a primary reducible inguinal hernia who were willing to participate in the study were included. Patients with bilateral hernias were offered repair on the most symptomatic side. Exclusion criteria were inability or refusal to give informed consent, alcohol or substance abuse, confirmed or suspected coagulopathy, ASA grade ≥III, and patients with suspected femoral hernias.

After having obtained written informed consent, patients were included either for the training intervention or the RCT. Study participants included in the RCT were randomly allocated to be operated on by an MD or an AC. The patients were operated on using an anterior mesh repair according to Lichtenstein, under local anaesthesia, using a commercially available lightweight polypropylene mesh^20,21^.

### Outcome measures

Before surgery, baseline assessment was done, including assessment of demographic data, assessment of the presence of co-morbidities, physical examination, assessment of inguinal pain, and assessment of health-related quality of life. After surgery, the study participants were followed up with a questionnaire and clinical assessment at 2 weeks, 1 year, and 5 years to assess the safety and effectiveness of the surgical training intervention and of task sharing for inguinal hernia mesh repair^16^. The 5-year follow-up data included information regarding self-assessed health score, groin pain, general health, and physical examination. Groin symptoms were evaluated using the Inguinal Pain Questionnaire (IPQ), with a score from one (no pain) to seven (severe pain). General health was assessed using a health thermometer scale ranging from 0 (the worst imaginable health) to 100 (the best imaginable health)^22^.

The primary investigator (PI) who carried out physical examinations at 2 weeks and 1 year after surgery, and who was blinded with regard to who performed the surgery, also carried out physical examinations at 5 years after surgery. The study participants were informed about follow-up visits through radio discussion and frequent radio announcements on the local radio station, which is the main source of information dissemination. They were also contacted by phone or via next of kin or a village elder and informed to come for follow-up at a certain date at Kamakwie Wesleyan Hospital or at Connaught Hospital, the main tertiary governmental hospital in the capital Freetown. Study participants who could not be contacted or who could not travel to the hospital were seen at an alternative site, most frequently in their homes. To optimize the follow-up rate, two local research assistants who both had vast knowledge of the study area participated in identifying and contacting patients. In addition, two experienced commercial bike riders were part of the follow-up. For study participants who had died, information about the date and cause of death was obtained from their next of kin or, if unavailable, village heads, friends, and relatives. In such instances more than one source was interviewed. All those with recurrent inguinal hernias were offered reoperation free of charge. Information on time of reoperation, surgical technique, and postoperative results were collected through careful follow-up by the PI.

### Statistical analysis

Descriptive statistics are used to present patient and operative characteristics. Student’s *t* test was used for comparison of numerical means and Fisher’s exact test was used for comparison of categorical data. *P* < 0.050 was considered statistically significant. The median (interquartile range (i.q.r.)) is used to present data with a non-normal distribution. Missing data is indicated in the tables but not included in the statistical testing. Statistical analyses were carried out using STATA 18.0 (StataCorp LLC, College Station, TX, USA).

### Ethical considerations

Ethical approval was obtained before the start of data collection from the Sierra Leone Ethics and Scientific Committee (22.05.2017I and 022.03.2023) and the trial was registered in the International Clinical Trial Registry (ISRCTN63478884). Patients were enrolled in the study after giving written informed consent and they were compensated for transportation costs.

## Results

**Fig. 1:**
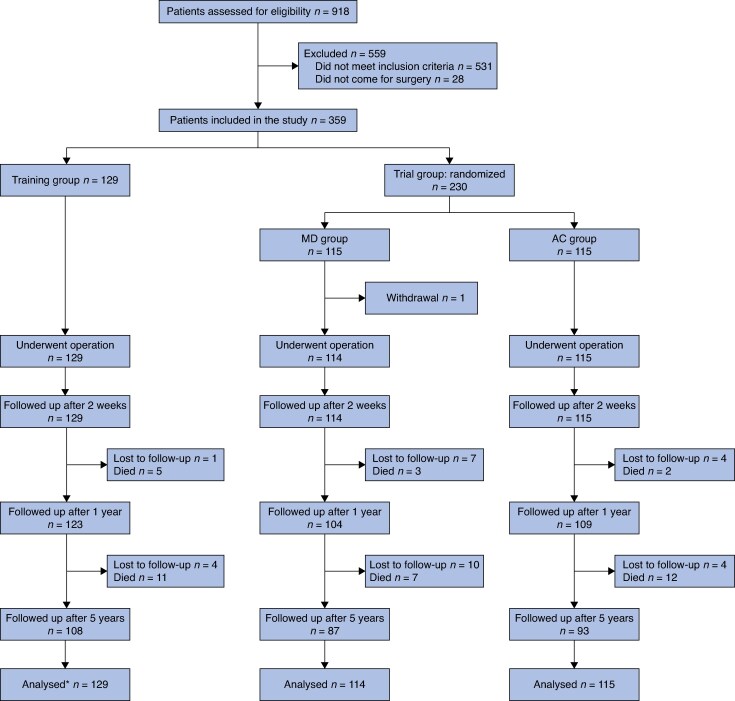
Flow chart of patients included in the training and RCT *Of the 129 training patients analysed, 72 procedures were performed for qualification. MD, medical doctor; AC, associate clinician.

A total of 918 patients were screened for the training and trial combined. Of those, 531 did not meet the inclusion criteria and 28 did not come for surgery, resulting in 359 patients being included: 129 for the training intervention and 230 in the subsequent RCT (*Fig. 1*). Patients included in the RCT were randomized for mesh inguinal hernia repair performed by an MD (115 patients) or an AC (115 patients). One patient from the MD group withdrew from the study before surgery.

This 5-year follow-up was carried out between April and June 2023. Of the 358 patients, 288 patients (80.4%) were alive, 40 patients (11.2%) had died, and 30 patients (8.4%) were lost to follow-up. Of the 288 patients who were followed up, 209 (72.6%) were seen at Kamakwie Wesleyan Hospital, 11 (3.8%) were seen at Connaught Hospital in Freetown, and the remaining 68 (23.6%) were seen at home or elsewhere. The median observation time for this group was 64.4 (i.q.r. 62.4–65.2) months. The mean age of patients who were lost to follow-up was lower compared with those who were not lost to follow-up (38.3 *versus* 43.9 years; *P* = 0.039) (*Table 1*). Patients with a scrotal hernia were more likely to be lost to follow-up compared with those whose hernia did not have a scrotal component (86.7% *versus* 47.0%; *P* < 0.001). Patients who were lost to follow-up had a slightly longer operating time compared with those who were not lost to follow-up (69.7 *versus* 59.6 min; *P* = 0.008). Of the 40 patients who died, 30 died between 1 year and 5 years after surgery. The mean age at surgery of patients who died was 52.1 years compared with 42.3 years for survivors. None of the deaths was related to the surgery or the inguinal hernia (*Table S1*).

**Table 1:** Perioperative characteristics of the patients who were not lost to follow-up *versus* those of the patients who were lost to follow-up

Characteristic	Not lost to follow-up (*n* = 328)	Lost to follow-up (*n* = 30)	*P*
Age (years), mean (95% c.i.)	43.9 (42.3,45.4)	38.3 (33.6,43.0)	0.039*
BMI (kg/m^2^), mean (95% c.i.)	21.8 (21.3,22.3)	21.0 (20.2,21.9)	0.351
**ASA grade**			0.444
I	150 (45.7)	11 (36.7)	
II	178 (54.3)	19 (63.3)	
Current smoking	138 (42.1)	16 (53.3)	0.252
Previous smoking	54 (16.5)	5 (16.7)	1.000
Scrotal hernia	154 (47.0)	26 (86.7)	<0.001*
IPQ score, mean (95% c.i.)	3.0 (2.9,3.2)	3.2 (2.7,3.7)	0.610
Self-assessed health score, mean (95% c.i.)	76.6 (75.0,78.2)	79.5 (73.7,85.3)	0.300
**Operation details**			
Operating time (min), mean (95% c.i.)	59.6 (57.5,61.7)	69.7 (61.6,77.9)	0.008*
Local anaesthesia only	325 (99.1)	29 (96.7)	0.296

Values are *n* (%) unless otherwise indicated. *Statistically significant. IPQ, Inguinal Pain Questionnaire.

At the 5-year follow-up, 18 study participants (5.0%) were found to have recurrent inguinal hernias after the index surgery (*Table 2*). Of those, 3 (16.7%) developed within the first month, 12 (66.7%) within the next 11 months, and 3 (16.7%) between 1 year and 5 years after surgery. The recurrence rates among the patients in the training group *versus* the trial group were 6.2% (8 patients) and 4.4% (10 patients) respectively. In the trial group, the recurrence rate was 1.7% (2 patients) in the AC group compared with 7.0% (8 patients) in the MD group (*P* = 0.059). Of the 15 patients who had a recurrent hernia within the first year after surgery, 13 (86.7%) underwent reoperation (either planned or as an emergency (9 and 4 patients respectively)). Eight reoperations were performed using the anterior mesh technique, three reoperations were performed using the Bassini or Nylon Darn technique, and two reoperations were performed using an unknown technique. Of the 13 patients who underwent reoperation, 3 patients had a re-recurrence at 5-year follow-up and all of these patients underwent anterior mesh repair that was performed by a specialist surgeon. Of the four emergency reoperations, only one was performed by a specialist using the Nylon Darn method. For the three patients who had a recurrent hernia between year 1 and year 5, only one patient was interested in a reoperation; the other two patients had no complaints and chose not to have further surgery. The details of the study participants with recurrent inguinal hernias are presented in *Table S2*.

**Table 2:** Outcomes at 5-year follow-up after mesh repair for an inguinal hernia

	Total (*n* = 358)	Training *versus* trial patients			Surgery performed by an MD *versus* an AC during the trial		
		Training (*n* = 129)	Trial (*n* = 229)	*P*	MD (*n* = 114)	AC (*n* = 115)	*P*
Death†	40 (11.2)	16 (12.4)	24 (10.5)	0.603	10 (8.8)	14 (12.2)	0.518
Recurrence†	18 (5.0)	8 (6.2)	10 (4.4)	0.458	8 (7.0)	2 (1.7)	0.059
**Wound healing‡**				0.779			0.113
Healed well	282 (78.8)	106 (99.1)	176 (98.3)		84 (73.7)	92 (80.0)	
Chronic wound	2 (0.6)	0 (0.0)	2 (1.2)		2 (1.8)	0 (0.0)	
Hypertrophic scar	2 (0.6)	1 (0.9)	1 (0.6)		1 (0.9)	0 (0.0)	
Missing	72 (20.1)	22 (17.1)	50 (21.8)		27 (23.7)	23 (20.0)	
**Scrotum‡**				0.023*			0.068
Normal	233 (65.1)	79 (61.2)	154 (67.2)		70 (61.4)	84 (73.0)	
Atrophy operation side	0 (0.0)	0 (0.0)	0 (0.0)		0 (0.0)	0 (0.0)	
Atrophy contralateral side	5 (1.4)	3 (2.3)	2 (0.9)		2 (1.8)	0 (0.0)	
Atrophy bilateral	1 (0.3)	1 (0.8)	0 (0.0)		0 (0.0)	0 (0.0)	
Hydrocele	48 (13.4)	25 (19.4)	23 (10.0)		15 (13.2)	8 (7.0)	
Missing	71 (19.8)	21 (16.3)	50 (21.8)		27 (23.7)	23 (20.0)	
**Contralateral side‡**				0.343			1.000
No inguinal hernia	177 (49.4)	60 (46.5)	117 (51.1)		58 (50.9)	59 (51.3)	
Status after successful repair	44 (12.3)	18 (14.0)	26 (11.4)		13 (11.4)	13 (11.3)	
Inguinal hernia	53 (14.8)	24 (18.6)	29 (12.7)		14 (12.3)	15 (13.0)	
Recurrent inguinal hernia	10 (2.8)	5 (3.9)	5 (2.2)		2 (1.8)	3 (2.6)	
Missing	74 (20.7)	22 (17.1)	52 (22.7)		27 (23.7)	25 (21.7)	
**Pain**							
IPQ score, mean (95% c.i.)	1.12 (1.07,1.16)	1.19 (1.10,1.27)	1.08 (1.03,1.13)	0.024*	1.03 (1.00,1.07)	1.12 (1.03,1.13)	0.100
Pain last week	28 (7.8)	17 (13.2)	11 (4.8)	0.012*	3 (2.6)	8 (7.0)	0.215
**Satisfaction with the result**				1.000			0.247
Satisfied	283 (79.1)	106 (82.2)	177 (77.3)		87 (76.3)	90 (78.3)	
Not satisfied	4 (1.1)	1 (0.8)	3 (1.3)		0 (0.0)	3 (2.6)	
Missing	49 (21.4)	22 (17.1)	49 (21.4)		27 (23.7)	22 (19.1)	

Values are *n* (%) unless otherwise indicated. *Statistically significant. †Including all patients in the analysis. ‡Observed during 5-year follow-up by health personnel. MD, medical doctor; AC, associate clinician; IPQ, Inguinal Pain Questionnaire.

At 5-year follow-up, 2 patients (0.6%) had a chronic wound and 2 patients (0.6%) had a hypertrophic scar. During physical examination on the contralateral side of the operation, it was found that 44 patients (12.3%) had successful surgery for an inguinal hernia, 53 patients (14.8%) had a current inguinal hernia, and 10 patients (2.8%) had a recurrent inguinal hernia.

**Fig. 2:**
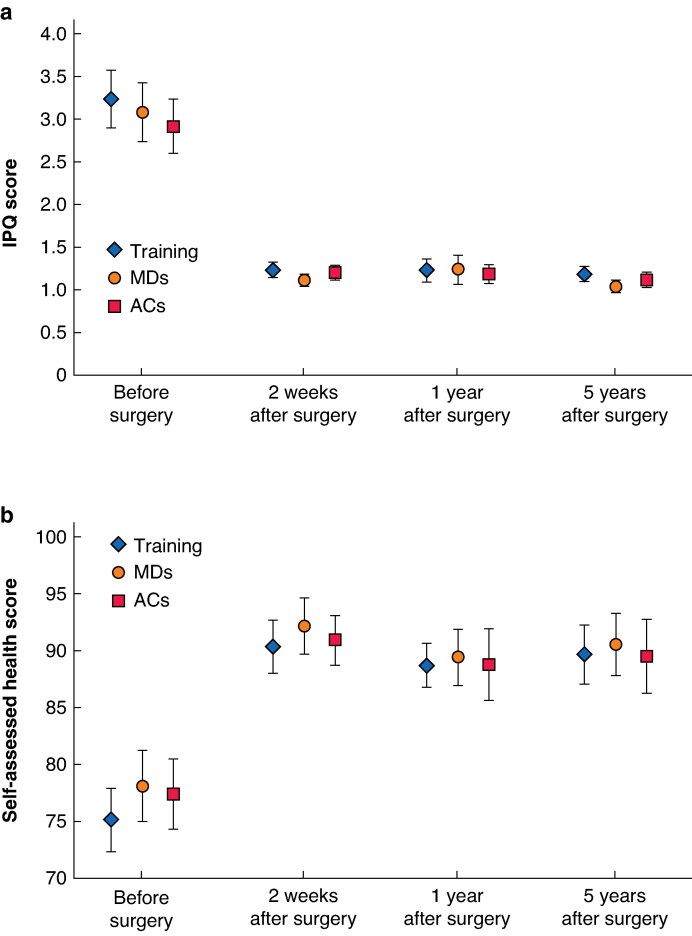
Scores for patients who underwent mesh inguinal hernia repair before and after surgery **a** IPQ score from 1 (no pain) to 7 (severe pain). **b** Self-assessed health score. Scores were collected before surgery and 2 weeks, 1 year, and 5 years after surgery. Surgeries were performed either during the training or during the randomized study by an MD or an AC. The self-assessed health score is based on a scale from 0 to 100, where 0 indicates the worst imaginable health and 100 indicates the best imaginable health. IPQ, Inguinal Pain Questionnaire; MDs, medical doctors; ACs, associate clinicians.

In total, 28 patients (7.8%) indicated that they had pain related to the surgery during the last week. The same patients also expressed that they used pain medication and had limitations in daily activities. Nevertheless, only four of the patients (1.1%) were not satisfied with the surgery, all due to complications (1 patient with wound infection, 1 patient with chronic pain, and 2 patients with recurrence). The mean IPQ score was 1.97 (95% c.i. 2.16 to 1.68) units lower compared with before the surgery and the mean health score increased by 13.1 (95% c.i. 10.8 to 15.4) on a scale from 0 to 100, where 0 indicates the worst imaginable health and 100 indicates the best imaginable health. The decrease in IPQ score and increase in self-assessed health score after surgery was comparable for all three follow-up rounds (*Fig. 2*). This impact was similar for surgeries performed during the training and during the RCT by MDs or ACs.

Hydrocele was more frequently observed for patients in the training group compared with patients in the trial group (19.4% *versus* 10.0%; *P* = 0.023). Training patients also reported more pain than trial patients, reflected by a higher pain score (1.19 *versus* 1.08; *P* = 0.024) and more pain during the last week (13.2% *versus* 4.8%; *P* = 0.012). For all other outcome parameters, no significant differences were observed between hernia repairs performed by MDs or ACs.

## Discussion

This 5-year follow-up study of patients who were operated on with mesh repair for inguinal hernias showed an overall recurrence rate of 5.0% and a mortality rate of 11.2%. In total, 7.8% of patients had pain related to the surgery during the last week.

The recurrence rate of 5.0% after 5 years was lower than the 13.1% recurrence rate of a nationwide population study in Sierra Leone, although in that study most surgeries were done without mesh and with a longer follow-up time^23^. Over 80% of the recurrences occurred within the first year after surgery. The recurrence rate of 5% observed in this study aligns with findings from a similar study conducted in Ghana, where non-specialist MDs and surgeons performed inguinal hernia mesh repair^24^. This consistency suggests that, with appropriate training and adherence to standardized surgical techniques, non-specialist healthcare providers can achieve recurrence rates comparable to those reported in specialized centres. Given the significant burden of inguinal hernias in low-resource settings, task shifting to trained general practitioners and non-specialist surgeons may represent a viable strategy to improve surgical access, while maintaining acceptable clinical outcomes. Additional research is needed to assess the long-term durability of such repairs, identify factors influencing recurrence, and optimize training protocols to enhance surgical proficiency and patient outcomes.

The mortality rate of 11.2% found at 5-year follow-up is notably high, but comparable to the study findings from Ghana, where a mortality rate of 12% was found^24^. None of the patients died in the first 6 months after surgery and none of the deaths could be directly attributed to the hernia repair. The mean age at surgery of patients who died before the 5-year follow-up was 52.1 years. The high mortality found in this study reflects more the life expectancy at birth for men of 59 years and the general status of the country with the triple burden of disease that includes infectious diseases, non-communicable diseases, and trauma^25–27^. In addition, lack of confidence in the health system, limited financial resources, insufficient healthcare provision at health facilities, and the role of traditional healers lead to delays in seeking care particulary in rural areas^28^.

Although inguinal hernia repair is amongst the most common surgical procedures performed and accounts for up to 16.0% of all surgeries in Sierra Leone, the unmet need remains high^29^. A cross-sectional study performed in Sierra Leone in 2020 found an incidence of groin hernia of 389 per 100 000 population per year, which is similar to the reported groin hernia repair rate. To reduce the backlog of unrepaired inguinal hernias, increasing the capacity of both MDs and ACs is highly cost-effective, but requires dedicated funding^23,30^.

At 5-year follow-up, patients in the training group reported a higher pain score compared with patients in the trial group and 13.2% of the patients in the training group reported pain during the last week. Even though this is higher than in the trial group, it is similar to chronic pain rates after Lichtenstein repairs reported in other publications^31,32^. The higher proportion of reported pain in the training group can be possibly explained by a lower self-assessed health score (74.5 *versus* 78.2; *P* = 0.026) before the surgery and a longer operating time (66.4 *versus* 57.2 min; *P* < 0.001)^16^.

Most of the inguinal hernia repairs in Sierra Leone are performed either by non-specialist MDs (39.7%) or ACs (30.3%)^29^. Even though the country has, since 2016, an established specialist training programme in surgery, task sharing will remain an important component to provide access to safe and affordable hernia surgeries, especially in rural areas. Systematic training for non-specialist MDs and ACs is a prerequisite to safeguard the quality of surgeries performed by these cadres.

Only 8.4% of the patients were lost to follow-up in this study due to a rigorous effort to find them by the data collection team. Almost one-third of the patients were seen outside of the study facilities (at home or elsewhere). Minimizing loss to follow-up is essential to obtain reliable results and minimize bias. Minimizing the number of patients who are lost to follow-up is particularly challenging in rural areas, where the population has a high rate of illiteracy, there is limited phone and internet access, and there is a poor road network. However, several strategies can be put in place to reduce loss to follow-up, including proper planning, radio announcements, use of local research assistants, and reimbursement of travel costs^33^. In this study, all of these strategies were used and, in addition, as the study area is a hard-to-reach area, the most effective means of transport is motorcycle, and the riders of commercial motorbikes have a comprehensive understanding of the territory. This method also helped to locate more study participants who had moved house and who are in places where radio coverage is absent.

Open anterior mesh inguinal hernia repair is the preferred technique compared with suture techniques for adults with groin hernias^34^. Currently, this is not standard practice in Sierra Leone due to the cost and the limited availability of mesh, along with the lack of widespread training in this technique. This situation is comparable to other sub-Saharan countries where resource constraints hinder the adoption of the most optimal evidence-based surgical techniques^4,35^. In addition to careful planning, educating patients and those who perform surgery, ensuring a supply of mesh at a low cost, and implementing a short-course in the technique for those who perform surgery would be a way to rapidly improve the quality of groin hernia surgery in Sierra Leone.

One of the main strengths of this study is the low number of patients lost to follow-up over the 5 years. In addition, patient questionnaires were combined with physical examinations to validate the information regarding hernia recurrence and other pathologies. All levels of data collection utilized local knowledge and resources, which were key to the successful implementation of this research. This study took place in a rural hospital, where most hernia surgeries take place in Sierra Leone, and therefore the results are generalizable to similar hospitals^4,33^.

A limitation of this study is that there were strict inclusion criteria for enrolment in the study and therefore study results are not generalizable to all patients with groin hernias. Hernia repair for women and children and emergency hernia repairs are all part of routine surgical practice. Future research should investigate the safety and effectiveness of task sharing in these instances.

Anterior mesh repair is the standard method for open repair of inguinal hernias worldwide and can be safely and effectively performed in low-income settings. Long-term outcomes after inguinal mesh hernia repair indicate that hands-on short-course training can be implemented effectively and that task sharing is safe and effective.

## Supplementary Material

znaf221_Supplementary_Data

## Data Availability

The primary data can be obtained from the corresponding author upon reasonable request.
