# Supplementary material for: Outcomes after elective inguinal hernia repair with mesh performed by associate clinicians versus medical doctors in Sierra Leone: 5-year follow-up of a randomized clinical trial
Source: Br J Surg. 2025 Dec 10;112(Suppl 15):xv50–7. doi: 10.1093/bjs/znaf221 (PMC12690658; doi:10.1093/bjs/znaf221)
Supplement: znaf221_Supplementary_Data [file znaf221_supplementary_data.docx]

**Outcomes After Elective Inguinal Hernia Repair Performed by Associate Clinicians vs Medical Doctors in Sierra Leone: Five-Year Follow-Up of a Randomized Clinical Trial**

Thomas Ashley^1,2^, Hannah F Ashley^2,3^, Andreas Wladis^4^, Pär Nordin^5^, Michael Ohene-Yeboah^6^, Isaac O Smalle^1,7^, Jessica H Beard^8^, Jenny Löfgren^9^, Hakon A Bolkan^2,10,11^, Alex J van Duinen^2,10,11^

^1^ Department of Surgery, University of Sierra Leone Teaching Hospital Complex (USLTHC), Connaught Hospital, Freetown, Sierra Leone

^2^ CapaCare, Masanga, Sierra Leone

^3^ The Lakes Medical Practice, Penrith, Cumbria, United Kingdom

^4^ Department of Biomedical and Clinical Sciences, Linköping University, Linköping, Sweden

^5^ Department of Surgical and Perioperative Sciences, Umeå University, Sweden

^6^ Department of Surgery, University of Ghana Medical School, Korle Bu, Accra, Ghana

^7^ Department of Surgery, College of Medicine and Allied Health Sciences, University of Sierra Leone

^8^ Department of Surgery, Lewis Katz School of Medicine at Temple University, Philadelphia, PA, USA

^9^ Department of Molecular Medicine and Surgery, Karolinska Institutet, Sweden

^10^ Department of Surgery, St Olav’s Hospital, Trondheim University Hospital, Trondheim, Norway

^11^ Institute of Nursing and Public Health, Norwegian University of Science and Technology (NTNU), Trondheim, Norway

**Corresponding author.**

Alex J. van Duinen, MD MIH PhD

Address: Institute of Nursing and Public Health

Norwegian University of Science and Technology (NTNU)

Postboks 8905, N-7491 Trondheim, Norway

Phone: +231 886083675; +47 93804555

Email: [aalke.j.v.duinen@ntnu.no](mailto:aalke.j.v.duinen@ntnu.no)

ORCID ID: 0000-0002-6943-0100

**Supplementary Figures and Tables**

| Supplementary Table 1. Mortality at 5-year follow-up after mesh repair for inguinal hernia | *pag. 2* |
| --- | --- |
| Supplementary Table 2. Hernia recurrence at 5-year follow-up after mesh repair for inguinal hernia | *pag. 3* |
|  |  |

# Supplementary Table 1. Mortality at 5-year follow-up after mesh repair for inguinal hernia

|  | Group | Camp | Age  (years) | ASA | Operation time (minutes) | Time after surgery (months) | Cause of death |
| --- | --- | --- | --- | --- | --- | --- | --- |
| 1 | training | II | 21 | 2 | 57 | 6 | Right lower limb swelling which gradually increased up the leg. Died within 3 days. |
| 2 | rtc – md | II | 36 | 2 | 34 | 6 | No information available |
| 3 | rtc – ac | III | 80 | 2 | 56 | 6 | Hypertension / hypertensive crisis. |
| 4 | training | II | 75 | 1 | 35 | 8 | Fell ill for one day and died. |
| 5 | rtc – md | II | 54 | 2 | 45 | 9 | Fall from palm tree prior to surgery, “chronic internal pain”. |
| 6 | rtc – md | II | 58 | 1 | 51 | 9 | Abdominal pain suspect for ulcer, admitted no surgery done and died 2 days later. |
| 7 | training | III | 37 | 2 | 55 | 13 | Hypertension / hypertensive crisis. |
| 8 | training | II | 37 | 1 | 42 | 13 | Acute pulmonary disease. Died before going to hospital. |
| 9 | rtc – ac | I | 50 | 2 | 48 | 13 | Snakebite |
| 10 | training | II | 67 | 2 | 58 | 15 | Unknown chronic disease |
| 11 | training | III | 65 | 2 | 44 | 29 | Died after 3 months with abdominal pain and swelling. |
| 12 | rtc – md | II | 35 | 2 | 46 | 31 | Hypertension and progressive eye disease. |
| 13 | training | I | 65 | 1 | 80 | 32 | Died after 6 months swelling right leg. |
| 14 | rtc – ac | III | 52 | 2 | 46 | 36 | Acute pneumonia. |
| 15 | rtc – ac | III | 56 | 2 | 35 | 36 | No information. |
| 16 | training | I | 78 | 1 | 60 | 36 | No information. |
| 17 | rtc – ac | II | 48 | 2 | 56 | 37 | Died during admission for abdominal pain and coughing. |
| 18 | training | II | 75 | 2 | 48 | 39 | Cough and weight loss, suspect for tuberculosis. |
| 19 | training | II | 52 | 2 | 70 | 43 | Died after short period of weakness. |
| 20 | rtc – md | II | 45 | 2 | 45 | 50 | Diagnosed with hypertension, leg infection, and depression. Died at home. |
| 21 | training | II | 38 | 2 | 54 | 50 | Died after 1 month with swelling left lower leg. |
| 22 | rtc – md | I | 40 | 2 | 73 | 51 | Died after period with icterus. |
| 23 | rtc – md | II | 70 | 2 | 51 | 52 | Was admitted with abdominal pain but died after discharge. |
| 24 | rtc – md | II | 45 | 2 | 60 | 52 | Collapsed after work on the farm and died shortly afterwards at home. |
| 25 | rtc – ac | III | 65 | 2 | 48 | 53 | Died after episode with dyspnoea. |
| 26 | training | III | 26 | 1 | 65 | 54 | Died after 1 year of complaints most suspect for colon cancer. |
| 27 | rtc – ac | III | 42 | 2 | 36 | 54 | Died after 1 week with abdominal pain and swelling. |
| 28 | rtc – ac | III | 65 | 2 | 42 | 55 | Chronic pulmonary disease. Admitted twice and died shortly after second admission. |
| 29 | training | II | 50 | 1 | 59 | 56 | Died at home after episode with abdominal pain, was unable to reach the hospital. |
|  | Group | Camp | Age  (years) | ASA | Operation time (minutes) | Time after surgery (months) | Cause of death |
| 31 | rtc – ac | I | 78 | 2 | 44 | 56 | Died after period of 3 months with chest and abdominal pain. |
| 32 | rtc – ac | III | 55 | 2 | 52 | 58 | Died after episode with urine tract infection and diarrhoea. |
| 33 | rtc – md | I | 43 | 2 | 73 | 59 | Died after 10 days with hiccups and abdominal pain. |
| 34 | rtc – ac | III | 60 | 2 | 68 | 60 | Died after period with one and of asthma episodes. |
| 35 | rtc – ac | II | 60 | 2 | 39 | 60 | Died after a short episode with coughing and weight loss most suspect for tuberculosis. |
| 36 | rtc – ac | II | 77 | 2 | 37 | 61 | Died after chronic episode with fever and body weakness. |
| 37 | training | II | 37 | 2 | 65 | 62 | Died at home after discharge and admission for episode with pneumonia. |
| 38 | rtc – ac | I | 38 | 1 | 60 | 63 | Fell from a tree and was admitted for 4 months and died at home after discharge. |
| 39 | rtc – md | I | 30 | 1 | 58 | 64 | Died at home after 2-3 days of sickness |
| 40 | training | II | 50 | 1 | 50 | 64 | Died after 3 years of chronic illness with abdominal distension and diarrhoea. |

ASA = American Society of Anaesthesiologists; rtc – ac = randomized controlled trial associate clinician group; rtc – md = randomized controlled trial medical doctor group.

# Supplementary Table 2. Hernia recurrence at 5-year follow-up after mesh repair for inguinal hernia

|  | Group | Camp | Age  (years) | ASA | Operation time (minutes) | Time after surgery (months) | Reoperated | Technique | 5 year outcome |
| --- | --- | --- | --- | --- | --- | --- | --- | --- | --- |
| 1 | rct – md | II | 57 | 2 | 80 | <1 | emergency | nylon darn | No recurrence at 1 yr follow-up, not found at 5 yr follow-up. |
| 2 | training | II | 60 | 2 | 72 | <1 | emergency | unknown | No recurrence at 5 yr follow-up. |
| 3 | training | II | 43 | 2 | 75 | <1 | planned | mesh repair | No recurrence at 5 yr follow-up. |
| 4 | training | II | 52 | 2 | 77 | 1 | emergency | unknown | No recurrence at 5 yr follow-up. |
| 5 | training | II | 52 | 1 | 61 | 4 | emergency | Bassini | No recurrence at 5 yr follow-up. |
| 6 | rct – md | III | 35 | 1 | 101 | 8 | no | n/a | Patient is symptomatic and waiting for surgery. |
| 7 | training | II | 65 | 2 | 67 | 12 | planned | mesh repair | Patient has a re-recurrent and is not interested in repair. |
| 8 | training | II | 55 | 2 | 134 | 12 | planned | mesh repair | Patient has a re-recurrent and is interested in repair. |
| 9 | rct – md | II | 38 | 1 | 63 | 12 | planned | mesh plug | No recurrence at 5 yr follow-up. |
| 10 | training | II | 70 | 1 | 85 | 12 | planned | mesh plug | No recurrence at 5 yr follow-up. |
| 11 | rct – md | II | 38 | 2 | 61 | 12 | planned | mesh plug | No recurrence at 5 yr follow-up. |
| 12 | rct – md | II | 45 | 2 | 68 | 12 | planned | unknown | No recurrence at 5 yr follow-up. |
| 13 | rct – ac | II | 66 | 1 | 68 | 12 | planned | mesh plug | Patient has a re-recurrent. |
| 14 | rct – md | III | 56 | 2 | 108 | 12 | no | n/a | Patient is asymptomatic and is not interested in re-operation. |
| 15 | rct – md | III | 52 | 1 | 72 | 12 | planned | Bassini | No recurrence at 5 yr follow-up. |
| 16 | rct – ac | III | 25 | 1 | 40 | 49 | no | n/a | Patient is symptomatic and waiting for re-operation. |
| 17 | rct – md | III | 39 | 2 | 71 | 58 | no | n/a | Patient is asymptomatic and is not interested in re-operation. |
| 18 | training | I | 68 | 1 | 74 | 62 | no | n/a | Patient is asymptomatic and is not interested in re-operation. |

ASA = American Society of Anaesthesiologists; n/a = not applicable; rct – ac = randomized controlled trial associate clinician group; rct – md = randomized controlled trial medical doctor group.
